# VR-Based Job Training System Using Tangible Interactions

**DOI:** 10.3390/s21206794

**Published:** 2021-10-13

**Authors:** Seongmin Baek, Youn-Hee Gil, Yejin Kim

**Affiliations:** 1 Contents Research Division, Electronics and Telecommunications Research Institute, 218 Gajeong-ro, Yuseong-gu, Daejeon 34129, Korea; baeksm@etri.re.kr (S.B.); yhgil@etri.re.kr (Y.-H.G.); 2 School of Games, Hongik University, 2639 Sejong-ro, Jochiwon-eup, Sejong 30016, Korea

**Keywords:** virtual training, job training system, VR environment, tangible interactions, depth data

## Abstract

Virtual training systems are in an increasing demand because of real-world training, which requires a high cost or accompanying risk, and can be conducted safely through virtual environments. For virtual training to be effective for users, it is important to provide realistic training situations; however, virtual reality (VR) content using VR controllers for experiential learning differ significantly from real content in terms of tangible interactions. In this paper, we propose a method for enhancing the presence and immersion during virtual training by applying various sensors to tangible virtual training as a way to track the movement of real tools used during training and virtualizing the entire body of the actual user for transfer to a virtual environment. The proposed training system connects virtual and real-world spaces through an actual object (e.g., an automobile) to provide the feeling of actual touch during virtual training. Furthermore, the system measures the posture of the tools (steam gun and mop) and the degree of touch and applies them during training (e.g., a steam car wash.) User-testing is conducted to validate the increase in the effectiveness of virtual job training.

## 1. Introduction

In job training, it is extremely important to provide job trainees with the opportunity to have a realistic experience within an unfamiliar work environment. Repetitive training is also important in job training; however, trainable work is limited, resulting in a lack of opportunity for accumulating skills. Furthermore, various difficulties are faced when training in a real-world environment, such as a limited physical space, facility management, safety accidents, and the costs of consumable materials during repeated training.

In recent years, virtual reality (VR) devices have become popular, and virtual environments have been applied to various fields such as games, education, medical care, and sports. VR has the advantage of being able to provide safe, repeatable, and specific training, focusing on improving social and practical skills required in the real world. Specifically, it can provide an effective job training environment through observations accompanied by direct interaction and experience with objects, rather than passive visual interaction-oriented virtual education [1,2]. Furthermore, the use of physical tools in virtual training can increase the effectiveness of training by improving the transfer and acceptance of learning [3].

For VR training to be effective during job training, it is important to provide realistic training situations. Hence, studies have been introduced to add appropriate physical objects for the virtual environment and use them for interaction with users to increase the immersion and situational awareness of the users [4,5,6]. Studies using haptic tools or hand tracking have also been actively conducted to enhance the interaction effect of the user [7,8,9,10,11,12,13]. Some studies have been published to improve the coexistence and mutual dependence between users and other objects in a virtual environment based on the presence of user avatars [14,15].

However, if only VR controllers are used as in conventional experience, it is difficult to touch objects directly, and an interaction is carried out through the action of in-air selection while holding the controller. This method does not support the awareness of real tools required in job training, making it difficult to implement a tangible interaction. For example, in mixed reality (MR) systems, physical objects can be controlled in a simple way such as translation and rotation and do not provide physical senses of touching. Moreover, this method facilitates only simple experiences in fields where training requires real contact (training using tools) rather than simple selections, and it is difficult to use at a level of actual applied force in training. Finger interaction methods have been proposed, which use glove-type haptic tools [16,17,18] to provide the feeling of grabbing a virtual object through force feedback. However, they are difficult to use in interaction-based content (tool-based job training), which requires exchange of force with the whole arm.

In this paper, we propose a VR-based job training system, in which users use real tools to provide tangible virtual training service. The proposed system provides a steam car wash training program to job trainees for safe, repeatable, and practical training. This system specifically uses a method of matching the vehicle model in a virtual environment with the coordinate system of the vehicle body in the real environment allowing the users to be trained while feeling an actual contact sensation when wiping with a mop in the virtual environment. Furthermore, to improve the immersion within the virtual environment, the proposed system virtualizes the user’s entire body by sending the 3D point and color data obtained from the calibrated Kinect sensor [19] to the content. A cost-effective problem may occur during virtual training for novice trainees because it requires an intensive intervention of the trained staff [20]. Therefore, this system was implemented to minimize expert intervention by providing information on the training through vision and audio within the virtual environment while proceeding with repeated training. The proposed method can increase the immersion and effectiveness through real-world-like training.

This paper is structured as follows. Section 2 analyzes previous related studies, whereas Section 3 introduces an overview of the system for tangible training. Section 4 then describes the construction of a reality-based virtual environment, and Section 5 details the tool-based user posture estimation method. Next, Section 6 shows the results of user experiments, and Section 7 provides some concluding remarks and introduces areas of future research.

## 2. Related Works

Various physical objects and devices have recently been used to increase the tangible sensation of the user. Simeone et al. placed physical objects corresponding to virtual objects and investigated the responses of the participants [4], whereas Lee et al. used a physical-virtual table to study the interaction with virtual characters and the physical effects [5]. He et al. proposed a design for the surrounding information to improve the safety problem and psychological discomfort and increase the situational awareness of physical elements [6].

Most VR content has been developed to be experienced with a controller held in the user’s hand for convenience and price issues. In recent years, research using haptic tools or hand tracking have been actively conducted to enhance the interaction effect. Loch et al. adopted physic components in the system during virtual training to allow users to know the feeling, size, and weight of the tools during training and proposed a haptic-interaction-based virtual training system [7]. Seo et al. proposed a method of learning through more intuitive interaction than learning through a VR controller by providing tactile sensations of air pressure and vibration through a fire extinguisher-shaped controller [8]. Arora et al. proposed VirtualBrics technology, a variety of controllers that facilitates a physical operation in a VR environment though LEGO-based tools [9]. Zhu et al. compared real tools and a VR controller to confirm whether the tangible interaction is the highest in real tools and developed haptic tools that were like real tools [10]. Shigeyama et al. developed Transcalibur, a portable VR controller that can render shapes by changing the mass properties on a two-dimensional plane to provide a realistic tool feeling [11]. Zenner and Krüger also proposed a technique for changing the drag and rotational inertia that the user feels by dynamically adjusting the surface area to deliver realistic and kinematic sensations [12]. Furthermore, VR and augmented reality (AR) based medical simulators are instruments for improving the skills of medical workers, and Talhan and Jeon conducted a study to provide various haptic effects by simulating physical effects through a pneumatic actuation [13]. Although many devices that track the user’s hand have recently appeared [16,17,18], the majority have modeled and visualized only a part (e.g., head or hand) of the user’s body for a VR interaction, thus lacking a feeling of realism.

In a study on the effect of user avatars on VR, Heidicker et al. presented a complete body model, which was mapped to the user’s movement and produced a higher coexistence and mutual dependence than the model composed of the user’s head and hand [14]. Franco and Peck reported that the representation of a user avatar in a virtual environment increased the subjective presence, increasing the validity of a stronger immersion and experience [15].

## 3. System Overview

**Figure 1:**
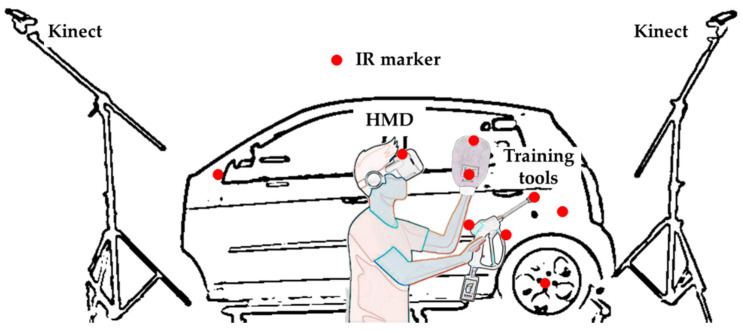
Overview of job training system in VR environment using multiple sensors.

The system proposed in this paper matches a real automobile body and a virtual vehicle model in the virtual environment by applying the infrared ray (IR) and depth sensors of a Kinect device to increase the effect of a tangible sensation. Furthermore, an IR marker is attached to a head mounted display (HMD) worn by the user to track the position and match the user’s position between the real and virtual environments. The user’s shape can be also seen in the virtual environment by extracting the user’s point and color values and sending them to the content for user visualization. Figure 1 shows the overall architecture of the proposed system.

**Figure 2:**
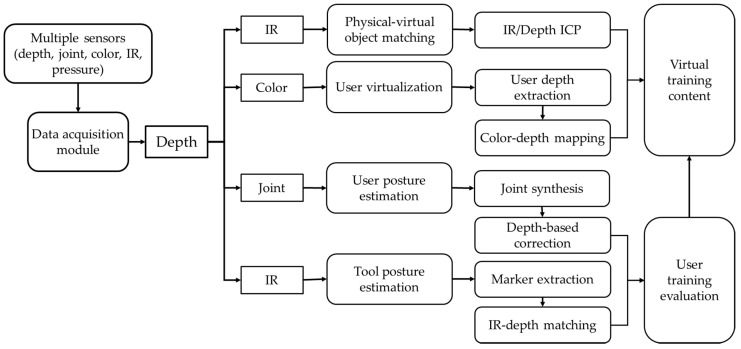
Overview of VR-based job training process.

For the tangible car wash training described in this paper, a real steam gun and a glove-type mob are used instead of a controller, and IR markers are attached to track the tool positions and directions. A button-type sensor is attached to the steam gun to check whether steam is being sprayed, and multiple pressure sensors are inserted in the mob to measure the pressure when wiping. The IR sensors for tracking the tools and multiple Kinect sensors for obtaining point and color data for the user-tracking are connected and applied. A Kinect is installed on both sides of the user, and after adjusting the direction for properly viewing the car and user, a coordinate system is matched through a calibration process. Figure 2 shows the overall system process proposed in this paper.

## 4. Construction of Physical Object-Based Virtual Environment

### 4.1. Multi-Camera Calibration

In this study, a calibration is required for each Kinect because multiple Kinect sensors are used for spatial recognition. To match the real and virtual spaces based on the depth, we used a depth point-based iterative closest point (ICP) algorithm [21]. The global coordinate system was set with the reference Kinect as the origin.

First, the incline of the Kinect sensors is calibrated for easier calculation. Points are extracted from the region corresponding to the ground among the depth data entered by each Kinect sensor, and a normal vector is found through the plane equation. The axis normal vector and the rotation matrix of the global coordinate system are then calculated to find the incline of the sensor. Next, the background and noise are removed from the input point data entered from Kinect, and only the image information that has undergone calibration is used as input data.

**Figure 3:**
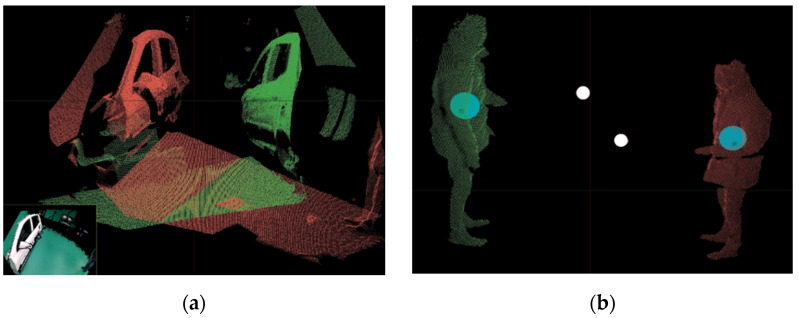
The 3D depth data visualization: (**a**) Captured from two Kinects (red points: left Kinect, green points: right Kinect; (**b**) Tracking of positions of a user and a capture tool in capture volume after background and noise removal (cyan circle: user position, white circle: capture tool position).

**Figure 4:**
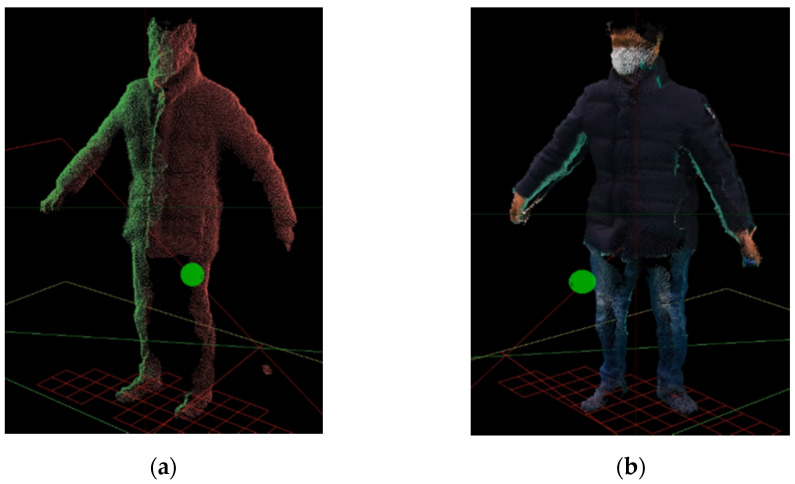
Multi-camera Calibration: (**a**) Aligned depth data from two Kinect inputs; (**b**) Color data result.

To find the depth point for calibration, we fabricated a capture tool by attaching a flat circle of 5 cm in diameter to a thin bar of approximately 50 cm in length. While the user moves inside the capture space, depth points (250 points) are obtained. Although the ICP algorithm requires a lengthy amount of time to find the matching relationships between points, with the proposed method, the matching relationships between points can be known immediately because the points obtained for each frame are input in sequential order. Therefore, R (rotation matrix) and T (translation matrix) values for different cameras are calculated quickly [22,23]. Figure 3 and Figure 4 show the calibration process and results for multiple Kinects. An average of 9 mm is measured from the depth points using the capture tool for the registration error.

**Figure 5:**
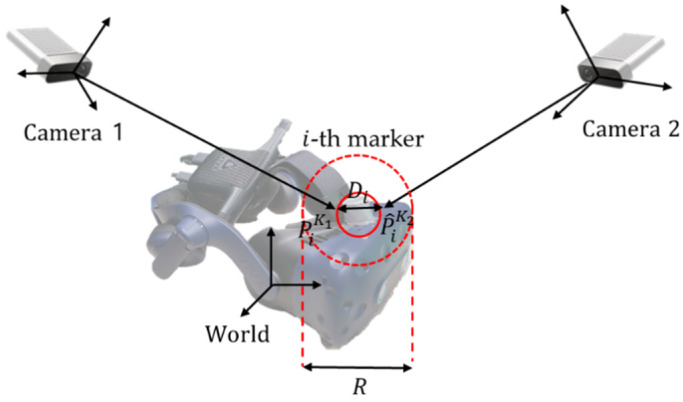
Error measurement between two Kinect captures: Here, $P_{i}^{K_{1}}$ and ${\hat{P}}_{i}^{K_{2}}$ are a marker position and a calibrated marker position in the world coordinate, respectively, for the i-th marker after slope correction of Kinects.

As shown in Figure 5, the error of two marker positions in the multiple calibrated Kinects, $K_{k}$, where $k\leq N_{k}$ and $N_{K}$ is the total number of Kinects, is calculated as follows:

(1) $$Error=\frac{\sum_{i=1}^{N_{i}}\frac{D_{i}}{R}}{N_{i}},$$

where i is the i-th marker, $N_{i}$ is the total number of markers, R is twice the marker size, and $D_{i}$ is the distance between the marker position, $P_{i}$, and the calibrated position, ${\hat{P}}_{i}$. after slope correction of Kinects.

In this study, the average error measured between the two Kinects (between $P_{i}^{K_{1}}$ and ${\hat{P}}_{i}^{K_{2}}$ in Figure 5) was 3.5 mm, which is less than a size of a pixel (4.8 mm). The resolution of the Kinect was 640 × 576 with a viewing angle of 75 × 65° at 30 fps, and the markers having a size of 20 mm were measured at an average distance of 2 m. Both calibration and registration errors are accurate enough to be used in virtual training.

### 4.2. Matching between Real and Virtual Objects

**Figure 6:**
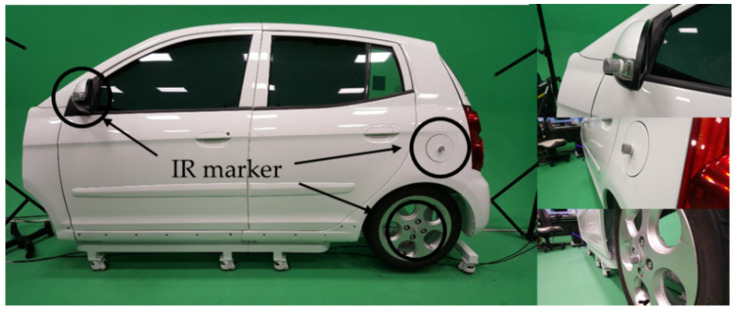
Real automobile body with IR markers.

**Figure 7:**
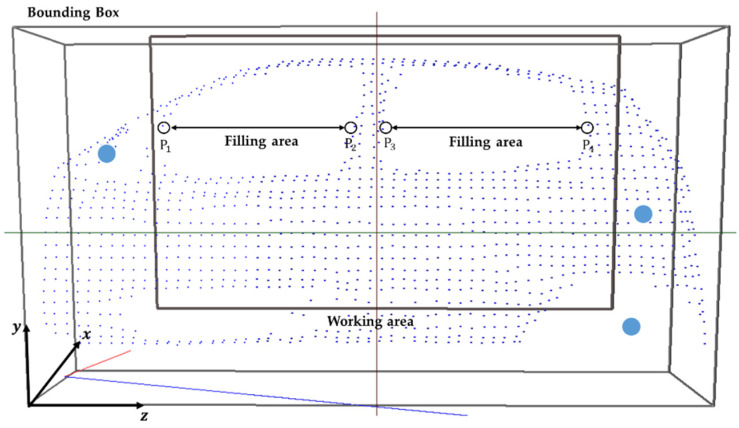
Sample points extracted from the automobile body and filling area on windows (cyan circle: IR markers).

In this study, we fabricated a virtual automobile model using a real automobile body, as shown in Figure 6, and thus the user can have the feeling of cleaning a real automobile. Because it was important to match the real automobile body with the virtual automobile model for a tangible interaction, we attached IR markers to the important points of the real automobile body (Figure 6) and extracted the points by setting the bounding box within the region where the automobile body exists in the depth data entered from multiple Kinects. Then, for a fast registration calculation of the real and virtual objects, we divided the bound box into voxels to calculate the average values of the points and conducted the sampling. Because only a side of the automobile body existed, we selected voxels having smaller values for the x-axis and removed the noise appearing in the backside. Figure 7 shows the IR markers attached on the automobile and the extracted sample points.

**Figure 8:**
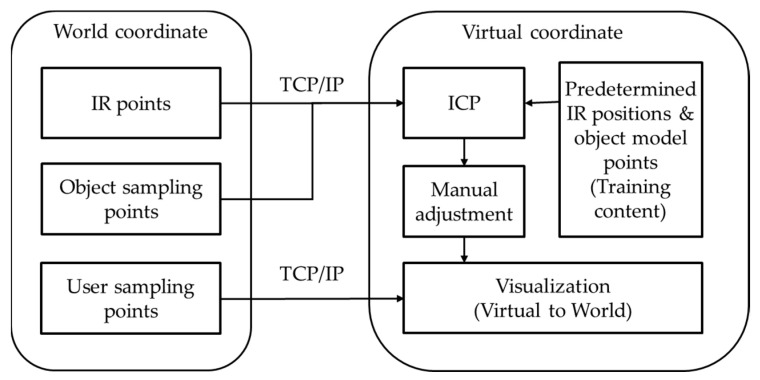
Process of sample points matching between real and virtual objects.

**Figure 9:**
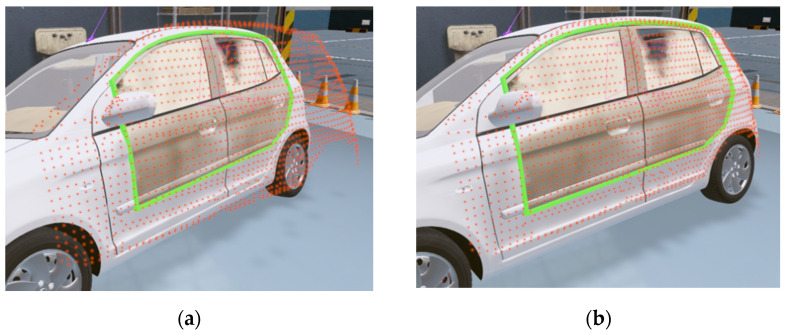
Sample points matched: (**a**) Before; (**b**) After.

When extracting depth data from a Kinect, a problem occurs in that the glass window parts of the automobile or the parts obscured by the curvature are not recognized. Because the interaction between the steam gun and the automobile points is used to check the degree of steam injection, or the contacting status is checked when wiping with a mop, we need a process for filling in the holes. Among the many hole-filling methods, this study proposes a fast and easy hole-filling method based on a grid-based method. As shown in Figure 7, the automobile region is divided into grids for sampling. While proceeding in the z-axis direction in the grid region, the region between the part where the points disappear ($\left[p_{1},p_{3}\right]$) and the part where they reappear ($\left[p_{2},p_{4}\right]$) is found and filled in with points. The point positions are determined by the interpolated positions between the first and second points ($\left[p_{1},p_{2}\right]$) and ($\left[p_{3},p_{4}\right]$). The IR marker positions, and the automobile’s sampling points are transmitted to the content to match the automobile body in the real-world coordinate system and the automobile model position in the virtual coordinate system. Because the automobile has a rigid body with a constant shape, it is easy to know which part of the virtual model to match. After extracting the required points from the automobile model in advance, the ICP algorithm is applied to arrange them. For some errors, the final positions were matched by applying a fine adjustment manually. Figure 8 shows the process used for matching the positions corresponding to the IR data and the virtual automobile model in the virtual coordinate system to the IR points and the automobile sample points in a real-world coordinate system. Figure 9 shows the matching result of the sample points between the real automobile and virtual automobile model.

**Figure 10:**
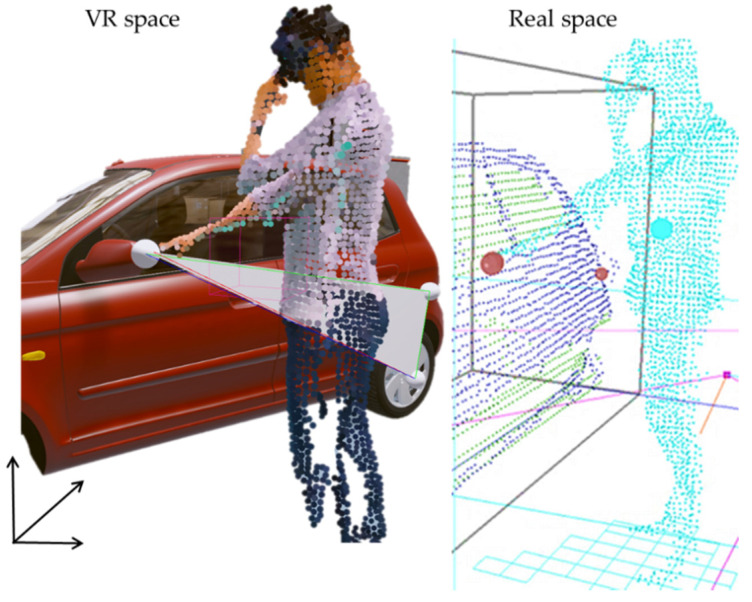
Visualization of a user in VR environment using sample points.

Furthermore, for visualization of the user in a virtual environment, the point and color values of the user model recognized by multiple Kinects are extracted, and the voxel sampling introduced earlier is applied to simplify the number of points of the user model (approximately 10,000 sample points are used) and thereby improved the visualization speed. The contents of the user model are virtualized by extracting the depth data and the corresponding color data, and the positions of the IR markers attached to the HMD are used to track the user position within the virtual environment. In this study, unlike conventional virtual experiences in which only partial body models are seen, a user can see a model that looks like him/herself in the VR environment, as shown in Figure 10, thereby increasing the immersion in virtual training, and providing an environment that facilitates the same interactions of the real-world job training.

## 5. Tool-Based User Posture Estimation

### 5.1. Tool Posture Estimation

**Figure 11:**
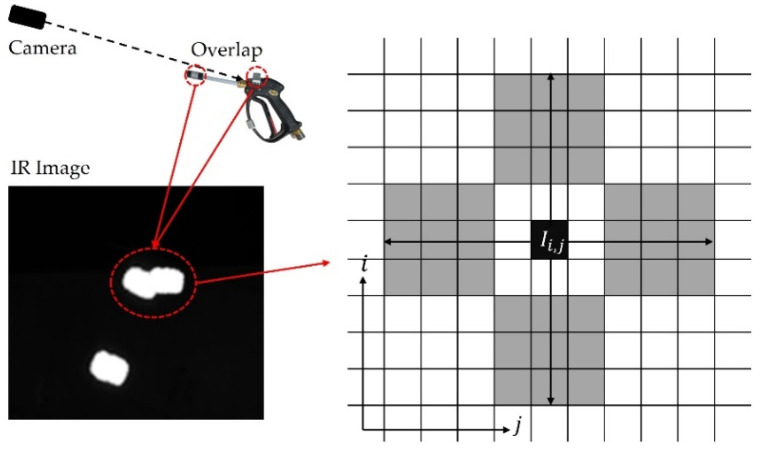
Estimation of the 3D marker position on the tool by searching the neighbor pixels around the center of the marker pixel on an IR image.

In this study, for tangible training, the user holds a steam gun in one hand and a mop tool in the other. For tracking each tool, IR markers are attached to the tools, as shown in Figure 11, and an IR sensor from a Kinect device is used for real-time tracking. In general, the three-dimensional (3D) position of each tool can be estimated through a triangulation-based stereo matching method based on images for tool tracking [24]. However, this method requires the simultaneous recognition from at least two RGB cameras and is sensitive to external noise. If the tool is occluded, it becomes difficult to estimate the position. Therefore, this study uses depth data to estimate the 3D positions of the tools.

First, after acquiring an IR image from the IR sensor, the marker’s center pixel information is found, as shown in Figure 11. In the depth map acquired at the same time as the IR image, the presence of depth data is confirmed through exploration in the up/down/left/right directions of the center pixel of the IR marker. By checking three pixels at a time in each direction, the range is gradually expanded. The search ends when points are exceeded, and the average value of the depth data in each direction is calculated. The 3D position by depth data can be obtained as follows:

(2) $$\begin{array}{l} z_{k}=d_{ij},\\ x_{k}=\frac{\left(pr_{x}-I_{i}\right)}{fl_{x}}z_{k},\\ y_{k}=\frac{\left(pr_{y}-I_{j}\right)}{fl_{y}}z_{k}, \end{array}$$

where pr and fl are the principal point and focal length of the Kinect, respectively, and $I_{i,j}$ is the pixel at (i,j) on IR image with a corresponding depth value, $z_{k}$, on the depth map (Figure 11). In this study, if each average is within the specified limit (40 mm), the average of the four directions becomes the final position; otherwise, they are recognized as different markers. As shown in Figure 11, even if two markers are in similar directions from the line of sight of the camera, different 3D positions can be obtained based on the difference in depth.

**Figure 12:**
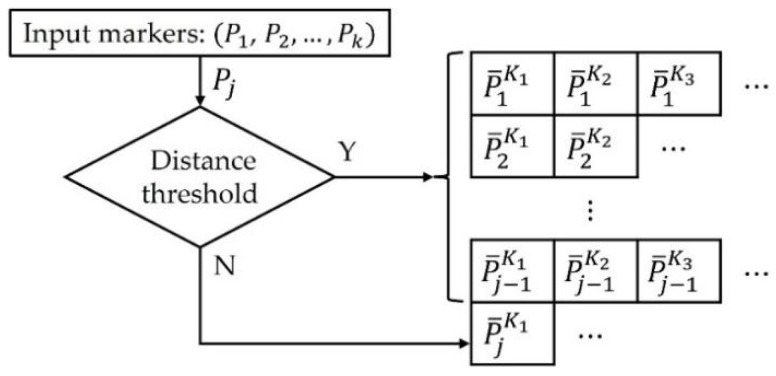
Classification of a marker position from multiple Kinects: Here, ${\bar{P}}_{j}^{K_{k}}$ is an average of j-th input marker positions from the k-th Kinect.

**Figure 13:**
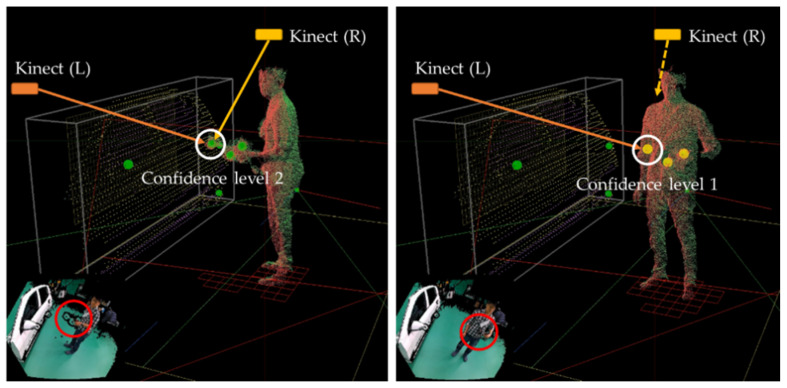
Examples of confidence levels based on marker positions (green circle: confidence level 2, yellow circle: confidence level 1).

In multiple Kinects, six (three for the steam gun, two for the mop, and one for the HMD) marker positions are tracked, and a pair of markers at a position within the error range based on the Euclidean distance is integrated into a single marker. If the 3D value input is close to a marker within the existing storage, it is input there; otherwise, it is input into a new storage. The new storage is moved to the existing storage, and the next marker value is tested. If the marker classification is completed, the positions of the markers in the same storage are integrated into the same marker position. If there are two or more marker positions in the storage, a value of 2 is assigned as the marker confidence level, and if there is only one marker position, a value of 1 is assigned. Because a depth map is used, even the 3D position of a marker seen by only one Kinect can be tracked. Figure 12 and Figure 13 show examples of the classification of markers and the confidence levels based on the positions of markers that can be tracked through multiple Kinects, respectively.

In this study, we also track the joint positions of the user together to prevent the recognition as a marker owing to the surrounding IR marker noise. For the posture estimation based on joint positions tracked, we utilized our capture system using multiple Kinects due to its robustness to external noises, real-time performance, and comparable accuracy to a commercial system (over 85%) [22]. A marker appearing around the head is classified as the HMD, and a marker appearing around the hand is classified as the steam gun or mop. Other distant positions are regarded as noises and removed. In the case of a steam gun, because the direction of the steam spray is important in the virtual environment, it is necessary to recognize the ID of the marker. The ID recognition is classified based on the marker’s distance. Three markers are attached to the steam gun, two on the mop, and one on the HMD. Among them, because the longest distance is set between the head and the MID marker, the head-MID marker is found first and the relationship with the TAIL marker in the surrounding area is then found to identify the direction of the steam gun. Among the remaining markers, a pair of markers at the distance corresponding to the mop becomes the mop position. If the marker is also successfully classified for each, the tracking state of each marker is set to on.

### 5.2. Tool Fabrication for Training

**Figure 14:**
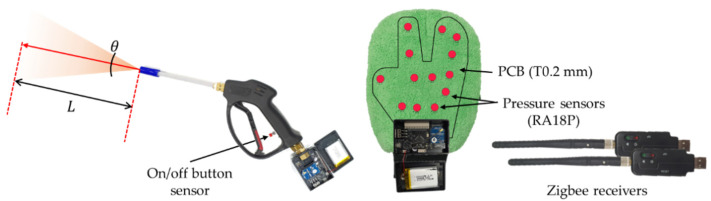
Training tools: Steam gun (L: distance, θ: direction) with a button sensor, mob with pressure sensors, and data transmitters for sensor connection.

In this study, we fabricated multi-sensor-attached training tools (steam gun and mop) to provide an environment like that of real-world training. A button sensor was connected to the steam gun to determine whether the steam is being sprayed, and pressure sensors were inserted in the mop to check how much force is used. A push-button (PBS-110) for checking the on/off position was used on the steam gun, and data were sent in 8 bits. A total of 13 pressure sensors (RA18P) were attached to the mop by fabricating a 0.2-mm PCB according to the glove shape. All pressure data are transmitted in 12 bits. Because the PCB is thin, the surface curvature of the automobile body can be felt, even if the pressure sensors are inserted into the mop. A wireless transmitter (Zigbee) is used for data transmission: pressure sensor data are sent in 20-ms intervals, and steam gun data are sent in 10-ms intervals. Figure 14 shows the fabricated training tools.

## 6. Experimental Results

**Figure 15:**
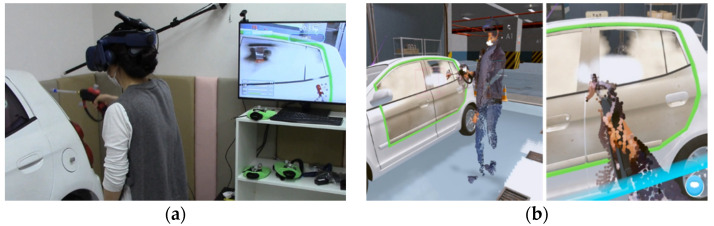
Steam wash system: (**a**) Virtual environment; (**b**) Training content.

The system used in this study consists of the following: a personal computer (with an Intel i7-9700 3-GHz CPU and an nVidia RTX2080 GPU) was connected to two Kinect sensors (Microsoft Azure Kinect DK) [19] and two data transmitters (Zigbee) of the training tools (steam gun and mop). As shown in Figure 15, the users participated in job training through the steam car wash training content created using a wireless HMD (VIVE Pro) [25] and the Unity game engine [26]. The accompanying video is shown in Supplementary.

### 6.1. Training and Evaluation Method

In the real-world environment, the steam gun should be used carefully, and it is dangerous to spray steam toward one’s body or at a very close distance from the car. Therefore, the training content should determine the spraying direction of the steam and the user’s body position to inform the user of a dangerous situation. To this purpose, the training content created in this study uses the direction of the steam gun, the steam spraying distance, and the user’s depth data to recognize a dangerous situation and provide a voice warning. Furthermore, we measured and evaluated the training performance of the user by composing training scores with four items: steam spaying (i.e., steam, whether steam was evenly sprayed on the car), steam distance (i.e., distance, whether steam was sprayed at a close range from the car), wiping (i.e., wipe, whether the steam sprayed was evenly and properly wiped), and user safety (safety, whether steam was sprayed to the user’s body). The “steam” and “wipe” were scored based on a method for adding points starting from zero based on how much of the region was filled. The scores for two items related to safety were based on a method of point deduction, i.e., the score decreased when the situation was deemed unsafe. Finally, the amounts of time spent on the steam spraying and wiping procedures were measured together.

**Figure 16:**
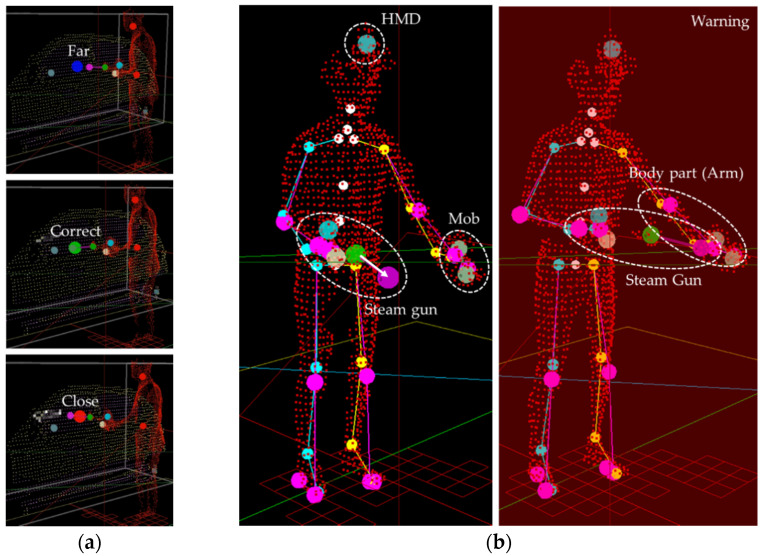
Evaluations of steam spray: (**a**) Distance (blue circle: far, green circle: correct, red circle: close); (**b**) Direction (red warning: dangerous situation).

As shown in Figure 14, if the steam gun is pointed toward the sampling points of the automobile body within the predefined region, the intersection position is calculated according to the distance (L) and direction (θ) of the steam gun. In the training content, the spraying distance and direction of steam can be set. Based on this, “steam” is evaluated based on the degree of interaction with the sampling points of the automobile body, and points are added only when the steam gun is within an appropriate distance (set to 20–35 cm as in the case of an actual steam car wash) after pressing the button. For example, if the distance between the intersection points and the steam gun is too far (more than 35 cm), the steam is not allowed to be applied to the surface of the automobile when sprayed, and a voice warning informs the user that the steam gun is too far away. If the steam gun is too close to the car (within 20 cm), information on being too close to the vehicle is provided, and at the same time, the score for the distance to the car is reduced. Furthermore, if the user is spraying steam toward his/her body, the user’s safety score is reduced, and a voice warning is given. Figure 16 shows examples of the evaluation according to the steam spraying distance and direction.

**Figure 17:**
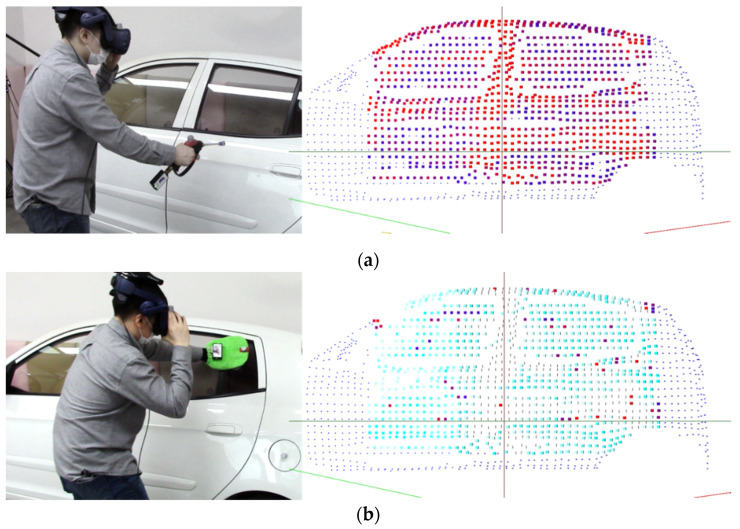
Changes in the sampling points: (**a**) Stem-spraying; (**b**) Mop-wiping.

During wiping training, the user holds a mop in one hand and wipes the car within the automobile region, and contaminants are wiped only when the mop position is close to the automobile model and the input signals arrive. When wiping, only when the position of the mop is within the predetermined distance from the actual automobile body and the user wipes with a certain pressure or higher is a point given. If the user wipes too weakly or too strongly, a voice warning is provided. In the case of pressure data, because each user has a different hand size, and it is difficult to press all pressure sensors while wiping, the highest-pressure value among 13 values is used for the calculation. Figure 17 shows the changes in the sampling points after steam-spraying and mop-wiping on the surface of the automobile body.

**Figure 18:**
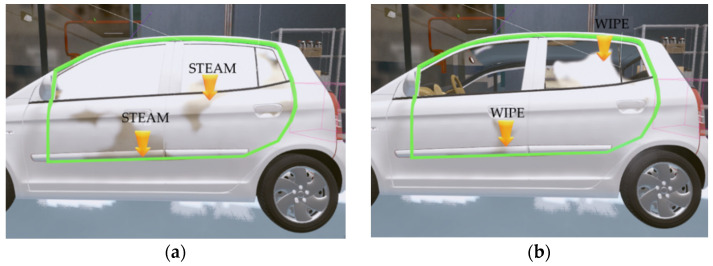
Indication of areas with low scores: (**a**) Stem-spraying; (**b**) Mop-wiping.

After the steam gun spraying is finished, the system tells the user which part of the automobile lacks steam spray. For such part, the work is divided into a 7 × 5 area, and each area with a low score is selected in ascending order and indicated with an arrow, as shown in Figure 18. For wiping training, the parts that have not been properly wiped are also indicated with arrows. When all training procedures are completed, the effectiveness of the training is measured through the final scores and the time performed. The system used in this study can save the training evaluation result for each user and thereby accumulate the data required for analyzing the training effect.

### 6.2. User Test

**Figure 19:**
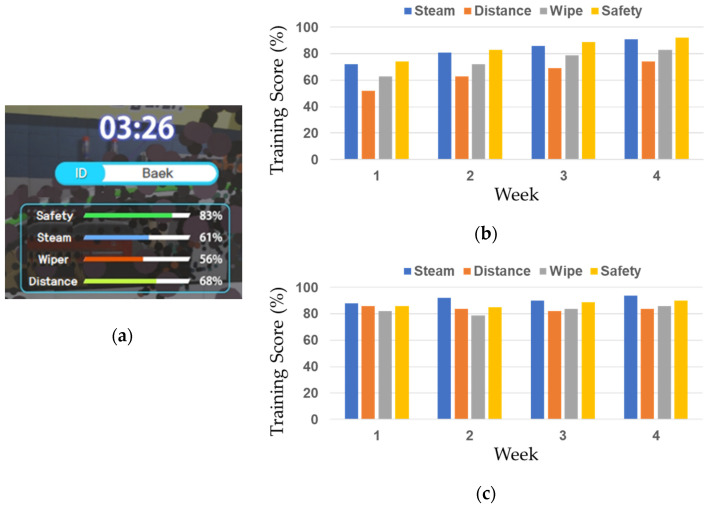
User case results: (**a**) Training scores; (**b**) Tangible interactions; (**c**) VR controllers.

To verify the usefulness and effectiveness of the job training content created through this study, we measured the work proficiency improvement for 12 participants (university students majoring in engineering.) The participants underwent the steam car wash training in both tangible interaction and VR controller environments 2–3 times per week for four weeks, and the average training scores of the four items (steam, distance, wipe, and safety in Figure 19) and time were recorded.

In the tangible interaction environment, the work proficiency of the participants continued improving during the evaluation, as shown in Figure 19. Relatively high scores were recorded for steam spraying (steam) and user safety (safety), which implies that the participants properly recognized and followed the warnings of the content. The participants found that wiping was more difficult than steam spraying, and it was most difficult to maintain a proper distance for the latter. In the case of wiping, relatively more sensors were attached to satisfy the real-world-like sophisticated tasks, and every participant was required to be trained on maintaining the optimal posture for achieving an appropriate spraying distance in a 3D space, like a real-world environment. Finally, the measured work time was 4–5 min during the first week, and afterward was reduced to 2–3 min.

In a VR-controller based environment in which a real automobile frame and car washing tools were not used, relatively high scores were observed, and no noticeable improvement was observed in terms of the work proficiency during the evaluation period, as shown in Figure 19. This was because, in the case of a VR controller, physical changes such as force control and posture correction were not specifically required because the car washing could be conducted through relatively simple interactions. In the case of the work time, the work was overall finished relatively quickly, typically within 1–2 min.

## 7. Conclusions

An intuitive interaction with a virtual environment is needed to increase the effectiveness and efficiency of virtual training. In this study, we developed a virtual training system using real objects to improve the tangible interaction during virtual environment-based training. The proposed system tracks the real training tools by using multiple sensors to increase the immersion during user training by matching the real-world environment with a virtual environment, visualizes the virtual space by matching it with the user’s real-world space, maps the real and virtual objects to provide a realistic physical touching sensation, and uses the system to propose a training evaluation method.

In this study, we improved the effectiveness of virtual training through user tests and obtained a positive effect for tangible interactions. By using a sensor-attached sprayer and mop, we observed that more effective training can be experienced compared to simple VR or MR experiences using conventional controllers because the users can determine how much force to apply based on the information provided on the actual contact and pressing forces.

The training system used in this study applies a real object (automobile frame.) Therefore, to use various shapes of virtual models, shapes of real models are required, despite the use of a virtual training environment. If a wall-shaped object with a similar tactile sensation is created instead of a real automobile, the cost of constructing the training environment can be reduced, and various training models can be provided. In addition, we can rely on the 3D printing devices to fabricate necessary objects in a cost-effective way.

Although only one user can currently be trained in the virtual environment, in the future, we plan to conduct a study to analyze the movement of each user when two or more people are in the same space and provide interactions for collaborative training. We plan to predict the user behavior more precisely by expanding the training evaluation and analyzing the starting position, as well as by moving the direction of the tools when spraying the steam or wiping the automobile.

## Data Availability

The datasets generated and/or analyzed during the current study are available from the corresponding author upon reasonable request.

## Supplementary Materials

The experiment results can be seen on the video located at https://drive.google.com/file/d/1NKdrjXmZbbR8hVqwRylM2faRofsWWO8E/view?usp=sharing.
